# Sex Hormones and Immune Dimorphism

**DOI:** 10.1155/2014/159150

**Published:** 2014-11-17

**Authors:** Aruna Bhatia, Harmandeep Kaur Sekhon, Gurpreet Kaur

**Affiliations:** Immunology and Immunotechnology Laboratory, Department of Biotechnology, Punjabi University, Patiala, Punjab 147002, India

## Abstract

The functioning of the immune system of the body is regulated by many factors. The abnormal regulation of the immune system may result in some pathological conditions. Sex hormones of reproductive system are one of the major factors that regulate immune system due to the presence of hormone receptors on immune cells. The interaction of sex hormones and immune cells through the receptors on these cells effect the release of cytokines which determines the proliferation, differentiation, and maturation of different types of immunocytes and as a result the outcome of inflammatory or autoimmune diseases. The different regulations of sex hormones in both sexes result in immune dimorphism. In this review article the mechanism of regulation of immune system in different sexes and its impact are discussed.

## 1. Introduction

Immune system is one of the crucial body systems that guard the body against infections and can be modified with genetic and environmental factors that may lead to certain pathogenic conditions. In the past few years, a significant increase in the studies related to immune system and its functioning has been noticed. But still the mechanism of regulation of immune system by hormones is not very clear. From the literature, it is apparent that immune system interacts with most of our body systems [1] and the system that modulates the immune system the most is the reproductive system [2, 3]. The interaction of reproductive system with that of immune system is attributed to the sex hormones and their hormone receptors on immune cells [4]. The regulation of immune response is different in males and females due to the presence of different hormones. In males it is testosterone that plays a major role as sex hormones and in females the predominant role is that of estrogen and progesterone. Due to menstruation females have periodic variations of sex hormones [5]. Female hormones have strong influence on the production and functioning of immune system cells and molecules. This difference in hormone levels in both sexes leads to immune dimorphism. This is the reason for the production of more vigorous immune response in females than in males [6]. The sex hormones also regulate the functioning of molecules of immune system as estradiol is reported to be one of the regulators of immune molecules like cytokines [7].

## 2. Immune System

Immune response is divided into two categories: nonspecific and specific. Nonspecific immune response is the innate or natural immune response that acts as the first line of defense against infections and recognizes structures like microbes. The components of nonspecific immune response are monocytes, macrophages, natural killer (NK) cells, dendritic cells, and granulocytes: neutrophils, eosinophils, and basophils. These cells attack microbes by phagocytosing them (neutrophils, monocytes, and macrophages), by lysis of infected cells (NK cells), or by producing cytokines to enhance nonspecific immune and specific immune responses [8]. Dendritic cells together with monocytes and macrophages act as antigen presenting cells (APCs). They take up foreign antigens (including viruses or pathogens), process them, and present antigen peptides on their surface for specific immune system mainly helper T lymphocytes [6].

**Figure 1:**
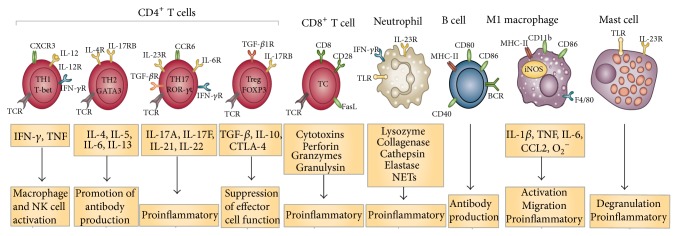
Cells and molecules of specific and nonspecific immune system [48, 49].

The specific immune response is divided into two types, that is, cell mediated and humoral immune response. Cell mediated immune response does not include antibodies but active immune cell population, namely, phagocytes, antigen specific T lymphocytes, and various cytokines, whereas humoral immune response is mediated by macromolecules found in extracellular fluid such as antibodies. T lymphocyte population is divided into cytotoxic T lymphocytes (Tc cells) that kill foreign or infected cells and helper T lymphocytes (Th cells) that provide help to other immune cells by producing cytokines. These Th cells are further divided into subtypes, that is, the Th1 subset producing interferon (IFN) gamma that promotes cellular immune responses, the Th2 subset producing mainly interleukin-4 (IL-4), IL-13, and IL-5 to aid humoral immune responses, and the Th17 subset producing IL-17, which plays a crucial role in autoimmunity and allergen-specific immune responses. The third division of T lymphocytes is regulatory T cell (Treg) that is centre of immunoregulation and is capable of suppressing both Th1- and Th2-mediated specific immune responses [9]. The components of immune system are shown in Figure 1.

## 3. Sex Hormones and Immune System

**Figure 2:**
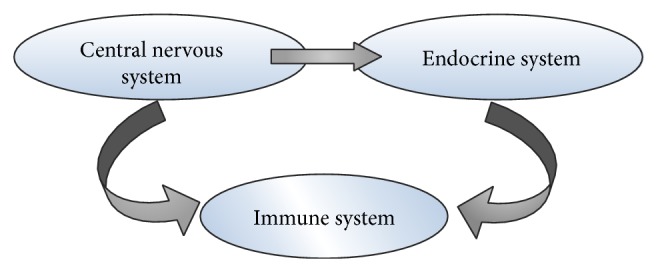
This figure shows the influence of different systems on the immune system. There is a bidirectional influence between the immune and CNS and immune and endocrine system (particularly gonadal) and endocrine (gonadal) and CNS. Sex hormones act on the CNS, macrophage/monocyte system, or the immune system itself, to affect immune responses [25].

Immune system does not work in isolation as immune, endocrine, and central nervous systems are integrated through a network of signal molecules (cytokines, hormones, and neurotransmitters) that act on a common set of receptors. It is influenced by many other factors that may be neuroendocrine peptides, sex hormones, or other metabolites. The interaction of these systems is shown in Figure 2.

**Figure 3:**
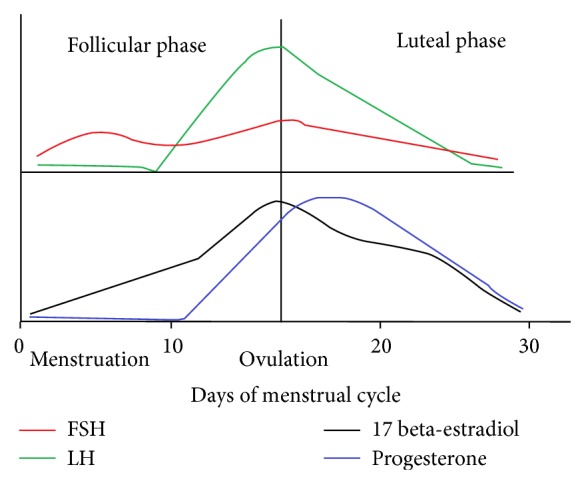
The menstrual cycle has 2 phases, namely, follicular phase and the luteal phase. The follicular phase starts at the beginning of menstruation. Follicle stimulating hormone (FSH) rises and stimulates follicular growth. The 17 beta-estradiol produced by the follicles inhibits FSH. 17 beta-estradiol continues to rise due to the growing dominant follicle and high concentrations of this hormone trigger the production of luteinizing hormone (LH) by the pituitary. LH then induces ovulation. After ovulation, the luteal phase starts. The remainder of the follicle develops into the corpus luteum, which starts producing progesterone and 17 beta-estradiol. If pregnancy does not occur, progesterone and 17 beta-estradiol concentrations decrease, menstruation starts, and a new cycle can begin [50].

Previous studies show the role of sex hormones in immune system functioning [10]. The difference in the concentrations and the type of the sex hormones in male and females during the life time brings alterations in the outcome of immune responsiveness. Sex hormones are divided into three classes: androgens (mainly testosterone), estrogens (mainly 17 beta-estradiol in the ovarian cycle), and progesterone. In males plasma testosterone concentrations remain stable almost up to 60 years of age and decline with aging. But in females there is a fluctuation in sex hormones estrogen and progesterone due to variation in concentrations of pituitary luteinizing hormone (LH) and follicle stimulating hormone (FSH) during menstrual cycle [11]. These variations include increase in 17 beta-estradiol and low progesterone plasma concentrations during follicular phase and high plasma 17 beta-estradiol and progesterone concentrations in the luteal phase, respectively [12]. Relation between hormonal level and menstrual cycle is shown in Figure 3.

### 3.1. Sex Hormone Receptors on Immune Cells

Sex hormones affect the immune system by increasing the number of circulating immune cells. Sex hormones either affect proliferation/apoptosis of the cells or induce production of new cells from the bone marrow. Sex hormones are steroids that are lipophilic in nature which facilitate their diffusion through cell membrane. This makes them affect the genetic material in the cell directly. Some workers described nongenomic effects of steroid hormones which are regulated via membrane receptors for these hormones on immunocytes [13]. Due to their lipophilic nature, sex steroids are able to alter membrane properties of immune cells by integrating into their membrane. This integration changes the function of immune cells. In different studies it is described that intracellular estrogen receptors are present in T lymphocytes and B lymphocytes [14], dendritic cells [15], and monocytes [16], in humans. As established by various studies activated lymphocytes (during pregnancy) can upregulate progesterone receptors. The presence of androgen receptors on human monocytes, neutrophils, NK cells, dendritic cells, or Treg cells is not confirmed in any study [17].

Transcription of a number of genes is regulated by steroid hormones by interacting with intracellular receptors, which are modular proteins composed of a ligand binding domain, a DNA binding domain, and several transactivation functions distributed along the molecule. Regulation of gene expression by hormones involved an interaction of the DNA-bound receptors with transcription factors which was also studied to be mediated by coactivators and corepressors. Depending on the nature of these interactions, the final outcome could be induction or repression of transcription [18, 19].

Effect of hormones on the immune response has been shown due to their effect on differentiation and maturation of immunocytes. Determination of the effect of antiestrogens on differentiation and maturation of dendritic cells was carried out by some workers. The authors observed the differentiation inhibitory effect of nonsteroidal antiestrogens (toremifene and tamoxifen) in the cultures of immature CD1a-positive DC in vitro from CD14-positive monocytes in the presence of interleukin-4 (IL-4) and granulocyte macrophage colony-stimulating factor. In the presence of antiestrogens the cells lost CD14 expression but remained CD1a-negative and have less dendritic processes than immature DC. Functionally, antiestrogen-treated cells were inferior to immature DC in inducing proliferation of allogeneic T cells and in producing IL-12 p70 protein after CD40 ligation. The expression of the costimulatory molecules CD80 and CD86 was differentially regulated by antiestrogens during DC differentiation. Antiestrogens were also able to inhibit the terminal maturation of DC [20].

While looking for the changes in the immune system during pregnancy an increased progesterone sensitivity of lymphocytes was observed which was due to activation-induced appearance of progesterone binding sites in the lymphocytes. Following recognition of fetus derived antigens *γ*/*δ* TCR+ cells developed progesterone receptors. Progesterone binding resulted in the synthesis of a mediator protein named the progesterone-induced blocking factor (PIBF). PIBF by acting on the phospholipase A2 enzyme interfered with arachidonic acid metabolism leading to induction of Th2 biased immune response and by controlling NK activity exerted an antiabortive effect [5, 21].

A subtype of estrogen receptor (ER) expressed in neutrophils from premenopausal women and in neutrophils from men under different estrogen conditions was identified in a study. The analysis was done on the association between the modifications in the expression of ER subtypes and neuronal nitric oxide synthase (nNOS) expression induced by estrogen. Neutrophils were isolated from premenopausal women during different stages of the menstrual cycle and from ten men for in vitro estrogen incubations. Outcomes showed that the neutrophils from premenopausal women expressed both ER-alpha and ER-beta subtypes which were increased in the ovulatory phase of the menstrual cycle. Neutrophils derived from men also expressed ER-*α* and ER-*β* but only ER-*α* expression was enhanced by in vitro incubation with 17*β*-estradiol (10^−8^ mol/L). In vitro incubation of neutrophils from women with 17*β*-estradiol enhanced expression of both ER-*α* and ER-*β* subtypes [22].

### 3.2. Impact of Sex Hormones and Immunocytes Interaction

Peripheral blood constitutes about 65% of the leukocytes: 32% granulocytes, 5–10% monocytes, and 30% lymphocytes. In females an increase in white blood cells counts was observed in the luteal phase of ovarian cycle and during pregnancy. Number of studies showed a decrease in the number of monocytes in follicular phase of cyclic females when estrogen level is high as compared to an increase in males and postmenopausal females [23, 24].

An experiment was made on orchidectomized PVG/c strain male rats. It was observed that orchidectomy potentiated the development of autoimmune thyroiditis induced by thymectomy and irradiation in rats. Administration of testosterone to orchidectomized rats reduced the development of autoimmune disease. When given in varying quantities by injection in oil over a period of 15 weeks the inhibitory effect on the development of both thyroiditis and thyroglobulin autoantibodies was found to be directly related to dose. Levels of testosterone between 150 ng and 150 *μ*g/100 gm body weight reduced the incidence and severity of the disease. Low doses of testosterone administered were also found to be beneficial to entire female rats. These results indicate that sex steroid hormones have an important modulatory influence on the genesis of autoimmune thyroiditis [25].

Changes in immunophenotypic expression of lymphocytes from thymus, bone marrow, spleen, and blood were analysed in 3- and 10-week-old male mice at periodic intervals following agonist administration. Upon agonist administration, plasma testosterone levels were significantly increased in prepubertal mice but were significantly decreased in postpubertal males. Absolute thymic weights, thymocytes, and T subsets were significantly increased from the third week regardless of gonadal status. Blood lymphocyte subsets showed a decreasing trend after agonist administration in prepubertal males, whereas no differences were observed in postpubertal males. No significant differences were observed in spleen cells after agonist administration. This study indicated that GnRH agonist effects on the immune system were independent of steroid hormone levels [26].

In one of the studies made to find out the effect of steroid exposure on immune function revealed that perinatal exposure to androgens transforms tissues like brain and genitalia permanently. The work showed the evidence suggesting a direct modification and regulation of immunocytes including Th1/Th2 balance through the interaction of androgen and estrogen and their receptors on developing immunocytes [27].

Apseloff et al. examined the clinically relevant effects of menstruation on leukocyte count. Healthy female subjects with normal menstruation were taken for a complete blood count with differential on days 1, 2, 7, 10, 17, 22, 25, and 32. Levels of luteinizing hormone and estradiol were measured on days 11 and 16 to determine the day of ovulation. Evaluation of the data revealed a trend toward higher leukocyte counts and absolute neutrophil counts at the onset of menses [28].

In order to determine the effect of the ovarian hormone cycle on immunity, immunoglobulin-secreting cell (ISC) frequency, and lymphocyte subsets in the blood, a study was made in healthy women. It was found that immunoglobulin A- (IgA-) secreting cells (IgA-ISC) were fourfold more frequent than IgG-ISC in peripheral blood mononuclear cells (PBMC). Further, the ISC frequency in PBMC was the highest (*P* < 0.05) during the periovulatory stage of the menstrual cycle. The work concluded that endogenous ovarian steroids regulate the ISC frequency [29].

Study was carried out to get insight into the sex differences of the basic nonspecific and specific immune responses. Intracellular types 1 and 2 cytokine production by stimulated male and female lymphocytes and monocytes in a whole blood preparation was measured by flow cytometry. The authors observed an increased percentage of interleukin-12 (IL-12), IL-1*β*, and tumor necrosis factor (TNF)-*α* producing monocytes and decreased percentage of IL-2 producing lymphocytes, that is, type 1 cytokine in men as compared to women. The results of the study suggested a gender difference in the balance between the specific and nonspecific immune response, that is, a more profound and higher state of excitation of the nonspecific immune response and relative suppression of the cellular immune response of the specific immune system in men as compared to women [30, 31].

The impact of different phases of menstrual cycle on the blood leukocytes was examined in twenty-four healthy women in their reproductive age group and having regular menstrual cycle during menstrual, proliferative, and secretary phases of the cycle. Total leukocyte count, absolute and differential counts of neutrophils, lymphocytes, and mixed cells (including eosinophils, basophils, and monocytes) were analyzed. The authors reported significant decrease in differential lymphocyte percentage in proliferative phase as compared to menstruation phase [32].

Variations in the blood leucocytes during different phases of menstrual cycle were observed in forty healthy women in the age group of 18–25 years with regular menstrual cycles of 30 ± 2-day duration. Total leucocyte count (TLC), absolute eosinophil count (AEC), and differential leucocyte count (DLC) were analyzed during the menstrual phase and proliferative and secretory phase during a single cycle. Results are as follows: the authors showed a statistically significant increase in TLC during and a significant increase in the neutrophil percentage during secretory phase. Lymphocyte count increased during proliferative and secretory phase [33].

## 4. Immune Dimorphism

It is apparent that females have better immune capabilities with higher immunoglobulin levels and stronger humoral and cell-mediated immune responses than males [34]. Studies showed their superior responses to a variety of antigens, their ability to reject allografts more rapidly, better in vitro response to mitogens and other in vitro immunologic assays, and relative resistance to the induction of immune tolerance [20]. Females tend to have a reduced incidence of certain tumors and generally resist a variety of bacterial and viral infections and parasitic infestations more successfully than males. Cytotoxicity to certain viruses is much greater in females than males also the survival rate of females is greater than males because of their better immune capability [35].

A large amount of information supports the fact that hormones of the endocrine system are involved in the immunological dimorphism in males and females. Major hormones included are growth hormone (GH), gonadal steroids, adrenal glucocorticoids, and prolactins. Complex hormonal interactions affect both developing lymphocytes and regulate mature immune cells. Hormonal interactions leading to immunological dimorphism are the effects elicited by estrogen and progesterone in female ovary after puberty. Effect of elevated estrogen levels to growth hormone secretion along with an increase in prolactins and thymosin release on the development of lymphocytes and stimulate immune cell functions in females was studied. Interactions of hormonal regulatory axes involving the hypothalamus, pituitary, gonads, adrenals, and thymus were also thought to be involved. Factors like IL-1 and IL-2 are elaborated by activated immune cells. Genetic components were also considered relevant under conditions of pathological disequilibrium leading to autoimmune disease [1, 36].

Difference in the vulnerability to autoimmune diseases in two sexes supports the sex based immune dimorphism. Women have been shown to develop diseases such as rheumatoid arthritis and multiple sclerosis as compared to men. More prevalence to autoimmune diseases in women was believed to be dependent on the influence of sex hormones. Authors believed that the autoimmune diseases development depends upon estrogen-induced immune suppression both in human autoimmune diseases and the experimental animal model counterparts [37].

Besides sexual differentiation and reproduction, sex hormones influence the immune system leading to immune dimorphism with females having higher immunoglobulin levels and mounting stronger immune responses than males. The greater immune responsiveness in females is also evident in their increased susceptibility to autoimmune diseases. Experimental studies reviewed described that normal mice show polyclonal B cell activation with increased expression of autoantibodies characteristic of autoimmune diseases by induction with estrogen treatment. Sex hormone levels in both humans and experimental models correlated with the activity of their cytokine-secreting cells indicating that sex hormones influence the cytokine milieu and suggesting that altered sex hormonal levels in autoimmune patients contribute to the skewed cytokine milieu characteristic of systemic lupus erythematosus [38, 39].

Clinical observations of a study indicate that some autoimmune diseases, such as rheumatoid arthritis and multiple sclerosis, frequently remit during pregnancy but worsen, or have their onset, in the postpartum period. The study was carried out in eighteen women with normal pregnancies in their third trimester and during the early postpartum period. It was reported that during the third trimester pregnancy, ex vivo monocytic IL-12 production was about 3-fold and TNF-*α* production was approximately 40% lower than postpartum values. At the same time, urinary cortisol and norepinephrine excretion and serum levels of 1, 25-dihydroxyvitamin D were 2 to 3-fold higher than postpartum values. Excessive production of IL-12 and TNF-*α* is linked to rheumatoid arthritis and multiple sclerosis. The study suggested that cortisol, norepinephrine, and 1, 25-dihydroxyvitamin D induced inhibition and subsequent rebound of IL-12 and TNF-*α* production may represent mechanism by which pregnancy and postpartum alter the course and susceptibility to various autoimmune disorders [6, 40].

Natural killer cells are CD3-negative, CD56-positive, and/or CD16-positive cytotoxic lymphocytes mediating first-line defense against various types of target cells without prior immunization. To assess the effect of the menstrual cycle and gender on NK activity work was done in which evaluation of 30 healthy women (mean age 28.1 years, range 21–39) in follicular and luteal phases, 29 postmenopausal women (mean age 58.8 years, range 42–72), and 48 healthy men (mean age 31.6 years, range 21–40) was done. The authors noted lytic units in flow cytometry and expressed the results as lytic units per 107 cells. Also progesterone levels were determined in the luteal phase of the menstrual cycle of healthy women by chemiluminescence assay. Results indicated higher NK cytotoxicity in the follicular than in the luteal phase of the menstrual cycle, and postmenopausal women and men showed NK activity similar to women in the follicular phase but higher than women in the luteal phase of the menstrual cycle. No correlation between NK activity and levels of progesterone was noted. The data suggest that progesterone does not influence NK activity directly and that other factors may explain the reduction of NK activity in the luteal phase of the menstrual cycle [41].

Gender has been a contributory factor in the incidence and progression of disorders associated with immune system. Evidence has suggested that gender may also play an important role in infectious disease susceptibility. It is well studied that females generate more robust and potentially protective humoral and cell-mediated immune responses following antigenic challenge than their male counterparts. The evidence for sexual dimorphism in innate immune responses to infectious organisms along with the recent studies providing mechanism underlying gender-based differences in infectious conditions such as bacterial sepsis is described and reviewed [40].

Rheumatoid arthritis (RA) is an autoimmune disease that is more common in women than in men. The peak incidence in females coincides with menopause when the ovarian production of sex hormones drops markedly. RA is characterized by production of proinflammatory mediators leading to the inflammation in the joint and eventually to bone loss. Animal studies have revealed distinct beneficial effects of estrogens on arthritis. A positive effect of hormone replacement therapy has been reported in women with postmenopausal RA. This review will focus on the influence of female sex hormones in the pathogenesis and progression of RA [42].

Due to the female predominance of autoimmune diseases, the role of gender and sex hormones in the immune system is of long-term interest. Estrogen's primary effects are mediated via estrogen receptors alpha and beta (ER *α*/*β*) that are expressed on most immune cells. ERs are nuclear hormone receptors that can either directly bind to estrogen response elements in gene promoters or serve as cofactors with other transcription factors (i.e., NFkB/AP1). Cytoplasmic ER and membrane associated ER impact specific kinase signaling pathways. ERs have prominent effects on immune function in both the innate and adaptive immune responses. Genetic deficiency of ER*α* in murine models of lupus resulted in significantly decreased disease and prolonged survival, while ER*β* deficiency had minimal to no effect in autoimmune models. The protective effect of ER*α* in lupus is multifactoral. In arthritis models, ER*α* agonists appear to mediate a protective effect. The modulation of ER*α* function appears to be a potential target for therapy in autoimmunity [43].

Work on the cyclical variation of total WBC count in women and men was done in a study. To undertook the study total of 56 girls and 74 boys had participated and blood sample was taken to count total and differential WBC count: for boys only one sample was taken and for girls three samples at different phases like first sample on 2nd or 3rd days of menstruation, second during proliferative phase (around 8th to 10th days), and third during secretory phase (around 22nd to 24th days). Statistical analysis of all the samples showed that among women there was a significant high total WBC count during proliferative phase compared to menstrual phase and secretory phase. There is a significant high total WBC count and lymphocytes in all the phases of women compared to men [44].

## 5. Cytokines and Sex Hormones

The correlation between serum levels of oestrogen, progesterone, and dehydroepiandrosterone sulphate (DHEA-S) and the number of human peripheral blood cells actively secreting interleukin-2 (IL-2), IL-4, IL-6, IL-10, tumour necrosis factor-*α* (TNF-*α*), or interferon-*γ* (IFN-*γ*) was determined in vivo. Simultaneous assessment of serum hormone levels and cytokine secreting cell activity throughout the menstrual cycle showed that the number of peripheral blood mononuclear cells (PBMC) able to secrete IL-4 in response to stimulation correlated significantly with oestrogen levels and fluctuated with the menstrual cycle in premenopausal women. The activity of IFN-*γ*-secreting cells, on the other hand, varied as a function of serum DHEA-S levels in premenopausal women. Number of cells secreting IFN-*γ* in men correlated with serum DHEA-S levels. In contrast, postmenopausal women had fewer cells actively secreting cytokines and the activity of these cells did not correlate with sex hormone levels. These results suggested that sex hormones may modulate cytokine production in vivo and contributed to gender-related differences in normal and pathological immune responses [45].

Changes in the cytokine milieu of patients with systemic lupus erythematosus (SLE) in association with abnormal levels of sex hormone levels in serum were examined in a study. The concentration of 17*β*-estradiol (E2), progesterone (Pg), and dehydroepiandrosterone-sulphate (DHEAS) was monitored in sera from 128 lupus patients and 96 controls and correlated with the activity of their cytokine secreting cells. Study revealed that SLE patients had significantly fewer cells secreting IFN-*γ*, increased serum E2 and Pg levels, and reduced serum DHEAS levels compared to normal controls [38].

A clinical study was conducted to evaluate the influence of gender on the release of proinflammatory and anti-inflammatory cytokines in the circulation and after lipopolysaccharide (LPS) ex vivo stimulation. The course of sex hormones in the acute phase response after surgery and assessed their correlation with cytokine release was also measured. The serum concentrations of interleukin-6 (IL-6), IL-8, IL-10, tumor necrosis factor-*α* (TNF-*α*), testosterone, estradiol, prolactin, procalcitonin, and sex hormone-binding globulin as well as the release of IL-6, TNF-a, and IL-10 after LPS ex vivo stimulation of whole blood in 26 patients without complications after major or minor abdominal surgery were measured. The study showed that therewas a gender-specific pattern of decreasing testosterone concentrations in men and increasing testosterone concentrations in women. Increasing estradiol concentrations were seen in both men and women. The ex vivo stimulated and systemic IL-6, IL-8, IL-10, and TNF-*α* cytokine release was not gender specific [46].

Role of inflammation in the development and progression of coronary heart disease (CHD) was studied by group of workers. For the study human monocyte-derived macrophages (HMDMs) were obtained from healthy normolipidemic men and postmenopausal women (age 50–70 years) and cultured in autologous serum along with both physiological and supraphysiological concentrations of estrogen or testosterone. HMDMs were stimulated with oxidized low-density lipoproteins, and the expression of the cytokines tumor necrosis factor *α* (TNF-*α*), interleukin-6 (IL-6), and IL-1*β* (IL1*β*) and of the acute-phase protein C-reactive protein (CRP) was measured. Both physiological and supraphysiological concentrations of testosterone reduced the expression and secretion of TNF-*α* and reduced the expression of IL-1*β* but did not affect the expression of IL6 or CRP. Estrogen caused a variable response in CRP expression that was positively associated with the plasma small dense LDL-cholesterol concentration of the donors [47].

## 6. Conclusion

Sex hormones have an impact on immune related diseases which results in immune dimorphism in males and females. Estrogen and testosterone are major regulators of immune system. The mechanism behind the sex hormone expression of immunocytes functions is attributed to the regulation through their interactions with the receptors on the immune cells which affects the production, maturation, differentiation, and ultimately the functioning of immune cells and molecules especially cytokines and development of immune related diseases. It is concluded that the modulation of cytokines production through hormones may help in future to design potential targets for therapy in sex hormones related inflammatory or autoimmune diseases.
